# From bark to bench: innovations in QS-21 adjuvant characterization and manufacturing

**DOI:** 10.3389/fimmu.2025.1677995

**Published:** 2025-12-02

**Authors:** Priyanka Talukdar, Peter H. Winegar, Graham A. Hudson, Maria C. T. Astolfi, Jamie L. Inman, Jay D. Keasling, Harshini Mukundan

**Affiliations:** 1 Biomedical Sciences and Bioengineering Department, Lawrence Berkeley National Laboratory, Berkeley, CA, United States; 2 California Institute for Quantitative Biosciences (QB3 Institute), University of California, Berkeley, Berkeley, CA, United States; 3 Biological Systems and Engineering Division, Lawrence Berkeley National Laboratory, Berkeley, CA, United States; 4 Joint BioEnergy Institute, Lawrence Berkeley National Laboratory, Emeryville, CA, United States; 5 Department of Bioengineering, University of California, Berkeley, Berkeley, CA, United States; 6 The Novo Nordisk Foundation Center for Biosustainability, Technical University Denmark, Lyngby, Denmark; 7 Department of Chemical & Biomolecular Engineering, University of California, Berkeley, Berkeley, CA, United States; 8 Chemical and Biological Technologies Programs, Office of National and Homeland Security, Lawrence Berkeley National Laboratory, Berkeley, CA, United States

**Keywords:** QS-21, adjuvants, synthetic biology, artificial intelligence, immuno-profiling

## Abstract

Adjuvants enhance immune responses; thereby increasing the efficacy of vaccines and longevity of the immune response. Despite this critical role, discovery of new adjuvants and pipelines to immunologically characterize and produce them at scale remain inefficient. In this review, we examine key challenges in the development of adjuvants and discuss emerging technological solutions using the saponin-based adjuvant, QS-21, as a central case study. QS-21 is a potent immunostimulant that promotes both humoral and cellular immunity and is a component of several FDA-approved adjuvant systems. In this manuscript, we review current understanding of the cellular and molecular mechanisms of QS-21 action, including interaction with antigen-presenting cells and role in inflammasome activation and T cell polarization. Despite its efficacy, factors such as hydrolytic instability, dose-limiting toxicity, and dependence on ecologically sensitive natural sources constrain broader application of this adjuvant. We discuss strategies to improve QS-21 function and delivery, including structural modification, combination with complementary immunostimulants, and formulation in nanoparticle-based systems; and address advances in synthetic biology and bioengineering that offer promise towards sustainable production of QS-21 and its analogs in microbial and plant-based platforms. Finally, we propose a vision for an integrated adjuvant development pipeline—from bark to bench—that leverages synthetic biology, artificial intelligence, and systematic immuno-profiling in order to accelerate discovery and deployment of next-generation adjuvants. Together, this review provides strategies to integrate and innovatively deploy emerging technologies in order to enable rapid discovery, development and deployment of known and new-to-nature adjuvants for health applications.

## Introduction

**Figure 1:**
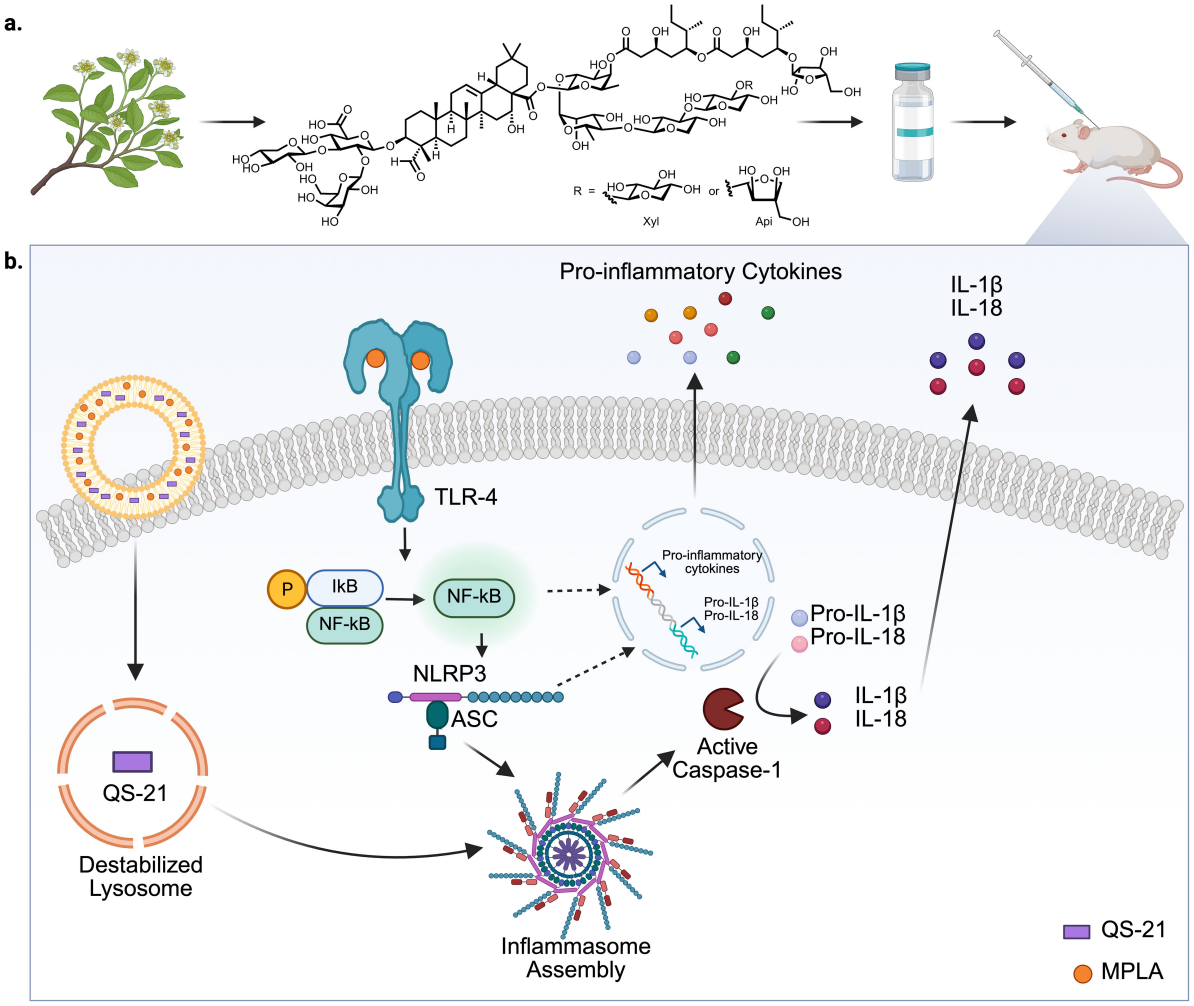
Schematic representation of the structure of QS-21 and QS-21-mediated activation of the NLRP3 inflammasome pathway. **(a)** QS-21 is a purified saponin usually extracted from *Q. saponaria* and has a central triterpene core, branched trisaccharide chain at C-3 position, a tetrasaccharide chain at C-28 position esterified to a glycosylated pseudodimeric acyl chain and the acyl chain is capped with a terminating xylopyranose or arabinofuranose sugar. **(b)** QS-21 and MPLA are co-delivered to antigen-presenting cells (APCs) via liposomes. MPLA, a Toll-like receptor 4 (TLR4) agonist, binds to TLR4 on the cell surface, initiating a MyD88-dependent signaling cascade that leads to IκB phosphorylation and degradation, allowing NF-κB translocation into the nucleus. This induces the transcription of pro-inflammatory genes, including pro-IL-1β and pro-IL-18. Simultaneously, QS-21 is internalized into the cell and activates the NLRP3 inflammasome complex, leading to the recruitment of ASC and activation of caspase-1. Caspase-1 cleaves pro-IL-1β and pro-IL-18 into active forms, which are subsequently secreted. Together, MPLA and QS-21 synergize to stimulate robust pro-inflammatory responses, contributing to the adjuvant activity of AS01 (7). Created with Biorender.com.

The discovery of adjuvants (i.e., alum, oil-in-water emulsions, CpG DNA, and QS-21) that enhance immune responses and their integration into vaccine formulations has enabled significant improvements in health outcomes. Still, progress in the discovery and use of adjuvants as immunomodulatory agents has been slow. Indeed, for over 70 years (since the 1930s), alum—a blend of various aluminum salts—was the sole approved adjuvant for human use. More recently, discovery of several other adjuvant candidates, and associated combinations have revitalized the field. Of these, the triterpene glycosides obtained from the bark of the Chilean soapbark tree *Quillaja saponaria* Molina (family Quillajaceae), a species native to central Chile (1), have been a primary focus of adjuvant research for more than 30 years, and have gained prominence due to their potent immunostimulatory properties (2). QuilA, a heterogenous crude aqueous extract from *Q. saponaria* bark has been extensively used in animal vaccines; however, toxicity of QuilA has prevented use in humans. Separating QuilA into different fractions *via* reverse-phase-high performance liquid chromatography (RP-HPLC) led to the identification of four immunomodulatory fractions: QS-7, QS-17, QS-18, and QS-21 (3), named for their species of origin (i.e., QS represents *Q. saponaria*) and relative retention time on RP-HPLC (i.e., 21 represents the 21st fraction in the RP-HPLC separation) (3). QS-18, the predominant saponin, was found to be the most toxic among the four fractions and demonstrated significant toxicity in animal models. In contrast, QS-7 and QS-21 exhibit lower toxicity, while QS-17 was more toxic than QS-21 (2–5). QS-21 being much more abundant than QS-7 (6), has been extensively studied for the past 25 years (6). QS-21 is a mixture of two isomeric molecules, each with four domains—the triterpene quillaic acid, a branched trisaccharide, a linear tetrasaccharide, and a glycosylated acyl chain and is currently incorporated in various vaccines such shingles, malaria, and others (**Figure 1a**). Whereas QS-21 offers extensive promise as an immunomodulatory adjuvant; it is important to recognize that the selection of the adjuvant candidate is based on its inherent favorable properties—low toxicity and abundance—that make it relatively easier to extract and use. In addition to QS-21, there are many saponins with adjuvant properties, including *Silene jenisseensis* saponins, *Momordica* subspecies saponins, saikosaponin a, and saikosaponin d (5). However, inherent properties of these saponins—low bioavailability/yield, high toxicity and others—preclude their broader use, limiting the range of adjuvants available for use. We contend that the use of disruptive technologies such as artificial intelligence and synthetic biology can enable tailored generation of new-to-nature adjuvants, allowing us to achieve the complete potential of saponin adjuvants for vaccine and countermeasure formulations.

The immunomodulatory potential of QS-21 is a major advantage over conventional adjuvants. Alum, for instance, primarily triggers an antibody-based T helper 2 (Th2) immune response (demonstrated by IgG1 production in mice) while providing limited stimulation of cell-mediated immunity (8, 9). In contrast, QS-21 enhances both antibody-mediated and cell-mediated immune responses, promoting a T helper 1 (Th1)-skewed immune reaction (10), inducing production of high levels of antibodies (IgG2a and IgG2b, along with Th2 antibody IgG1 in mice) and antigen-specific cytotoxic T lymphocytes (CTLs) (9). However, the precise molecular mechanisms of QS-21 mediated modulation of adaptive immunity remain incompletely understood. Understanding the immunological mechanisms of QS-21 mediated stimulation is essential for improving efficacy. This is also critical in order to enable tailoring of properties, which can enable holistic adjuvant foundries for use with new antigens and therapeutic candidates in the future. One key limitation to such characterization is the lack of systematic *in vitro* and *in vivo* pipelines for assessment of immunological function in a physiologically relevant manner, ensuring translation relevance for human use. This need for comprehensive immunological profiling methods and platforms, and curation of associated data for use with comprehensive computational analytics is the *first* opportunity we present in this review.

Despite the many advantages, broad usability of QS-21 as a stand-alone adjuvant has been restricted due to its limited availability, hydrolytic instability, isomeric heterogeneity, and dose-dependent toxicity (2, 3, 6). Importantly, QS-21 exhibits significant hemolytic toxicity; and studies show that the molecule can induce significant (~50%) hemolysis of sheep red blood cells at concentrations as low as 7–9 µg/mL (3, 11). The associated side effects restrict its dosage to approximately 50 µg, except in cancer patients, where doses of 100–200 µg are approved for use as higher doses have been found to generate improved antibody titers (6, 12). Because of these challenges, QS-21 has been incorporated into adjuvant systems—combinations of immune stimulators and delivery platforms—ensuring both safety and efficacy. For example, the Adjuvant System 01 (AS01) (**Table 1**) from GSK plc combines QS-21 with a Toll-Like Receptor 4 (TLR4) agonist monophosphoryl lipid A (MPLA) in a liposomal formulation, mitigating adverse effects while enhancing immunogenic properties (13). MPLA is a detoxified lipopolysaccharide derived from the rough mutant strain R595 of *Salmonella enterica* serovar Minnesota, obtained through hydrolysis of the C1 phosphate and (*R*)-3-hydroxytetradecanoyl groups (50). Different AS01 adjuvant formulations contain varying concentrations of QS-21 and MPLA, each optimized for specific vaccine applications. For example, AS01B (AS01 with 50 µg QS-21 and 50 µg MPLA, per injected dose) is a key component of vaccines such as Shingrix, Mosquirix (RTS,S malaria vaccine) (51). The Novovax coronavirus vaccine, NVX-CoV2373, on the other hand utilizes Matrix-M, an adjuvant system containing QS-21, QS-7, and QS-17 (52, 53). The chemical structure of QS-21 has also been modified to tune bioactivity, and synthetic analogs of QS-21 can exhibit improved stability and reduced toxicity (54–56). Combining QS-21 with other immunostimulants, such as TLR agonists, has also shown promise (57, 58). Together, these advancements overcome some of the current limitations and expanding utility of QS-21. However, the combinatorial use of QS-21 with other adjuvants currently has to be tailored to each use case. Thus, optimizing the adjuvant formulation for each vaccine can be time consuming and laborious. Understanding immunogenic profiles of QS-21 and other saponin adjuvants to enable more streamlined use of these candidates for immunomodulatory applications is the *second* opportunity space discussed in this article.

**Table 1:** List of all adjuvants mentioned in the paper.

Adjuvant name	Class	Active/Key components
AS01/AS01B/AS01E (13, 14)	Saponin/TLR-based (liposome formulation)	QS-21, MPLA
AS02A/AS02B/ AS02V (13, 15–17)	Saponin/TLR-based (oil-in-water emulsion)	QS-21, MPLA
AS03 (18)	Oil-in-water emulsion-based	*DL*-*α*-Tocopherol, squalene, polysorbate 80
AS04 (13)	TLR/alum-based	MPLA, aluminum salt (alum)
AS05 (19)	Saponin/TLR/alum-based	QS-21, MPLA, aluminum salt (alum)
AS15 (20)	Saponin/TLR-based	QS-21, MPLA, CpG ODN
CAF01 (21)	Synthetic liposome-based	Cationic lipid dimethyldioctadecyl-ammonium (DDA), glycolipid trehalose-6,6′-dibehenate (TDB)
Matrix-M (22)	Saponin-based (nanoparticulate complex)	Matrix A (saponin Fraction-A from *Q. saponaria*), Matrix C (Fraction-C-primarily QS-21)
ISCOM (22, 23)	Saponin-based immune-stimulating complex (antigen incorporated)	Saponins, cholesterol, phospholipids, sometimes viral glycoproteins
ISCOMATRIX (24, 25)	Saponin-based immune-stimulating complex (antigen free)	Saponins, cholesterol, phospholipids
Army Liposome Formulation (ALF) (26–28)	Saponin/TLR-based (liposome formulation)	QS-21, MPLA
Alum (29)	Aluminum salt-based	Aluminum salts
Alhydrogel (30)	Alum-based (wet gel suspension)	Aluminum hydroxide
Addavax (31)	Squalene-based (oil-in-water nanoemulsion)	Squalene
GPI-0100 (32)	Synthetic saponin-based	Semi-synthetic triterpenoid glycoside
QT-0101 (33)	Saponin-based	Deacylated QS-21 analog
CRL1005 (34)	Synthetic polymer-based	Non-ionic block co-polymer
TiterMax (35)	Water-in-oil emulsion-based	Squalene, metabolizable oil and block copolymers, microparticulate stabilizer
BCG-CWS (36–38)	Multi-pattern recognition receptor based	Cell wall skeleton (CWS) of Bacillus Calmette–Guérin (BCG)
CL401 (39–41)	TLR-based	8-hydroxyadenine (TLR7 ligand), Pam2C (TLR2 ligand)
Motolimod (VTX-2337) (42)	TLR-based	TLR8 agonist
MPLA (43)	TLR-based	Less toxic LPS derivative from *Salmonella minnesota* R595
3D(6-acyl)A-PHAD (44)	TLR-based	Synthetic MPLA
FP20 (45)	TLR-based	Small molecule glucosamine based TLR4 agonist
CpG ODN (46, 47)	TLR-based	Synthetic DNA with unmethylated cytosine phosphoguanine motifs—TLR9 agonist agonist
Poly I:C (48, 49)	TLR-based	Synthetic double stranded RNA—TLR3 agonist

A critical challenge in the large-scale production of QS-21 remains the reliance on natural extraction from the bark of *Q. saponaria* trees, raising concerns due to limited tree populations, and batch-to-batch variability, and supply-chain challenges during emergencies. Alternative methods for QS-21 production, including total chemical synthesis and semi-synthetic approaches, have been explored but remain technically complex, resource-intensive, and as yet, unable to meet global demand. A promising solution lies in the heterologous expression of QS-21 biosynthetic pathways in plant cell culture or engineered microbial cell culture. With further development, such systems could offer a scalable, controlled, and sustainable alternative to plant extraction, ensuring high purity and consistent yields while reducing environmental impact. They can also eliminate supply chain dependencies, ensuring self-reliance for adjuvant biosynthesis. Biomanufacturing QS-21 and other saponins in yeast-based systems at scale can greatly enhance access and usability of these natural products. Synthetic biology enabled solutions can help realize this possibility, and this is the *third* opportunity we present. As noted earlier, the advent of artificial intelligence and machine learning (AI/ML) has enabled the emerging ability to predict phenotype from genotype and expedite synthetic biology pipelines. Incorporation of such modalities into synthetic biology pipelines for adjuvant generation can enable new-to-nature adjuvants with tailored properties.

Our goal for this review is to provide a comprehensive description of the state of the art in the immunology (innate and adaptive), formulation, and manufacturing of QS-21; while assessing areas of emerging technological innovation that can expedite and revolutionize adjuvant science. Since most mechanistic studies of QS-21 have been conducted using AS01 or in combination with MPLA, we present QS-21 and AS01-related findings together to underscore their interconnected immunological mechanisms and provide a coherent understanding.

## Immunology

### Activation of innate immune response

The role of QS-21 in activating the innate immune response has been investigated across diverse model systems, offering valuable mechanistic insights, albeit with several limitations. Although it is not known if QS-21 directly interacts with any specific immune cell receptor, the oligosaccharide chain of saponins can target C-type lectin receptors (e.g., DEC-205) expressed on antigen presenting cells (APCs), which could contribute to enhanced uptake and presentation to T cells (59). In human peripheral blood mononuclear cells (PBMCs), AS01 has been shown to preferentially activate innate myeloid cells—monocytes and myeloid dendritic cells (mDCs) (60). In myeloid cells, both AS01 and QS-21 have been shown to stimulate the expression of surface markers Human Leukocyte Antigen–DR (HLA-DR), CD86, CD11c, and CD54 in monocytes, in monocytes, whereas MPLA was shown to induce only CD11c expression. In mDCs, neither AS01 nor MPLA stimulation has been associated with significant upregulation of surface markers, but QS-21 has been shown to induce modest increases in HLA-DR, CD86, and CD11c expression (60). These findings contrast with other studies in the literature wherein AS01 has been reported to activate monocyte-derived dendritic cells (moDCs), a key contributor to its adjuvant activity (61, 62). Analysis of cytokine responses revealed that both monocytes and mDCs produce IL-8 and TNF-ɑ in response to AS01 and MPLA, with monocytes showing a higher cytokine response than mDCs, whereas QS-21 by itself did not elicit cytokine production (60). These studies highlight the synergistic effects of QS-21 and MPLA, with QS-21 promoting upregulation of costimulatory marker expression and MPLA enhancing the cytokine response.

QS-21 has been shown to exhibit avidity to cholesterol, and can intercalate into cholesterol-rich regions of cell membranes (63). The adjuvant is endocytosed in a cholesterol-dependent manner, subsequently accumulating in lysosomes (64). Studies with human moDCs have shown that QS-21 can cause lysosomal destabilization by mediating membrane permeabilization, enabling translocation of antigens into the cytosol and promoting antigen cross-presentation (63–65). Lysosomal disruption triggers phosphorylation of Syk kinase, which is a crucial step for downstream NF-κB activation and inflammatory gene expression (64). Notably, lysosomal acidification has been shown to be essential for QS-21-induced cytokine responses, as inhibition of vacuolar ATPase with bafilomycin A1 significantly reduces IL-6 and TNF production (64). QS-21 also directly activates human moDCs, enhancing expression of surface markers (HLA-DR and CD86) and proinflammatory cytokines (IL-8, IL-6, and TNF) in a manner dependent on cathepsin B, a lysosomal cysteine protease (64). *In vivo*, the importance of cathepsin B extends beyond cytokine production, as its deficiency impairs QS-21-adjuvanted CD4^+^ and CD8^+^ T cell responses to hepatitis B surface antigen (HBsAg) (64). Collectively, cathepsin B and Syk represent key effectors in the lysosome-dependent immune activation pathway initiated by QS-21, bridging innate dendritic cell activation to robust adaptive, antigen-specific T cell responses.

Studies in mice have shown that QS-21 in combination with TLR4-agonist MPLA can induce ASC-NLRP3 inflammasome (a multi-protein complex crucial for innate immunity) activation and elicit caspase-1-dependent IL-1β and IL-18 release in mouse macrophages and DCs (**Figure 1b**) (66, 67), further emphasizing the role of lysosomal acidification in promoting inflammasome activation by QS-21. Mice with Caspase 1 or MyD88 knockouts exhibited decreased infiltration of monocytes (in caspase-1 knockout only), as well as reduced neutrophil accumulation in draining lymph nodes (DLNs) following QS-21 immunization, accompanied by diminished polyfunctionality of antigen specific CD4 and CD8 T cells (67). Caspase-1 activation can trigger pyroptosis (highly inflammatory programmed cell death), resulting in release of damage-associated molecular patterns (DAMPs) such as high mobility group protein B1 (HMGB1). Interaction of DAMPs with TLR4 and RAGE (receptor for advanced glycation end-products) can activate the innate immune response in a MyD88-dependent manner (68). QS-21 immunization can result in the production of HMGB1 in mice lymph nodes (LNs), and inhibition of HMGB1 has been shown to reduce antigen-specific CD4 and CD8 responses in QS-21 immunized mice, further emphasizing the importance of both Caspase-1 and MyD88 in mediating the adjuvant effects of QS-21 (67). However, the role of inflammasome in QS-21 adjuvanticity remains controversial. Some studies have reported that, *in vivo*, this signaling pathway may actually suppress antigen-specific responses as NLRP3 deficient mice exhibited enhanced antigen specific T and B cell responses following immunization with QS-21 and HIV gp120 (66). These conflicting reports in immunological findings further emphasize the need for systematization of approaches for characterization of adjuvant function in a manner that ensures physiological relevance and translation to human studies.

QS-21 injection in mice has been associated with colocalization in subcapsular sinus CD11b^+^CD169^+^ macrophages, a unique subset of APCs that play a crucial role in antigen retention and immune modulation (67). This finding underscored the importance of CD169^+^ macrophages in mediating the adjuvant effect of QS-21, as depletion of the LN macrophages led to suppressed DC activation and diminished antigen-specific T and B cells responses. CD169^+^ sinus lining macrophages in human LN slices also demonstrate highest capacity uptake of AS01-like liposomes (69). The subcapsular sinus macrophages, considered an essential reservoir of IL-18, have been reported to play an important role in orchestrating AS01-induced IFN-γ production by lymphoid cells (70). QS-21, whether isolated from plant cell culture or tree bark, has been shown to enhance immunogenicity of ovalbumin (OVA), trafficked to DLNs, and specifically targeted CD169^+^ macrophages in mice (71). Unlike other adjuvants such as alum or squalene-based formulations, QS-21 from either source induced a distinct reorganization of CD169^+^ macrophages, leading to their depletion and a subsequent influx of activated monocytes, neutrophils, and innate lymphoid cells (71). This restructuring can enhance recruitment and activation of DCs, creating a more favorable environment for robust T cell priming.

Beyond macrophages, DCs also play a pivotal role in antigen presentation to T cells and in driving their proliferation. Following varicella zoster virus (VZV) glycoprotein E (gE) immunization in mice, AS01 enhances recruitment of MHCII^high^ DCs in LNs, promoting DC activation and increasing number of antigen carrying APCs in LNs in comparison to immunization with gE alone (62). Depletion of DCs abrogated activation of antigen specific T cell responses in vaccines with AS01, highlighting a role for activation of DCs prior to activation of adaptive immune response. Further characterization of DC subtypes recruited to DLNs following immunization with AS01-adjuvanted VZV gE in mice revealed that the majority of the MHCII^high^CD11c^+^ migratory DCs were CD26^+^ conventional DCs (cDCs), predominantly cDC2s, which primarily activate CD4^+^ T cells (61). This was followed by cDC1s, specialized in antigen cross-presentation to CD8^+^ T cells, and a smaller population of inflammatory cDC2s (inf-cDC2s) (61). Inf-cDC2s represent a recently identified subset of pre-cDC-derived cells that possess hybrid features of cDC2s and monocyte-derived cells and rely on the chemokine receptor CCR2 for bone marrow egress (72). Notably, the authors emphasize a non-redundant role for inf-cDC2s in mediating the immunostimulatory effects of AS01, as *Ccr2^-/-^* mice exhibited impaired antigen-specific antibody production following booster immunization and generated suboptimal CD4^+^ and CD8^+^ T cell responses (61). In addition, CD103^+^ and CD8α^+^ DCs, critical for antigen cross-presentation, have also been shown to play an essential role in mediating adjuvant effects of AS01 (67). In mice lacking BATF3, a transcription factor important for the development of CD103+ and CD8a+ DC subsets, QS-21-adjuvanted antigens failed to elicit CD8^+^ T cell responses and showed diminished polyfunctionality of antigen-specific CD4^+^ T cells (67).

QS-21 and MPLA have been reported to act synergistically to promote IFN-γ production from natural killer (NK) and CD8^+^ T cells in DLNs, a response critical for DC maturation and the development of a Th1-biased adaptive immune response (70). Individually, QS-21 and MPLA have also been shown to induce expression of IFN-γ-related chemokines, CXCL9 and CXCL10, in the DLNs of HBsAg immunized mice (70). Supporting these findings, intraperitoneal injection of QS-21/MPLA in C57BL/6 mice acutely elevated serum levels of the inflammatory cytokine IL-6 and chemokine MCP-1 within 2 hours, and recruited neutrophils, monocytes, and DCs to the site of injection (73). In a complementary study using human LN slices, liposomal QS-21 was shown to directly stimulate production of proinflammatory cytokines like IL-1β, IFN-γ, and IL-18 (69).

Taken together, these studies underscore the multifaceted role of QS-21 in activating the innate immune response through diverse and complementary mechanisms. QS-21 stimulates human DCs and monocytes by directly upregulating activation markers and proinflammatory cytokines, through lysosomal destabilization and cathepsin B-dependent pathways. In murine models, QS-21 triggers activation of the inflammasome, induces caspase-1-mediated pyroptosis, and promotes the release of DAMPs like HMGB1, which further amplify innate immune signaling via TLR4 and MyD88. Moreover, QS-21 interacts with subcapsular sinus macrophages, which are essential for DC activation and antigen presentation. Its synergistic effects with MPLA in AS01 further amplifies innate immune responses by promoting IFN-γ production and driving a Th1-skewed adaptive immune response. Still, there are some contradictions around the role of QS-21 and MPLA and their synergistic immunomodulation of innate immunity, further emphasizing the need for a more systematized and comprehensive mechanistic understanding of these processes.

### Activation of adaptive immune response

**Figure 2:**
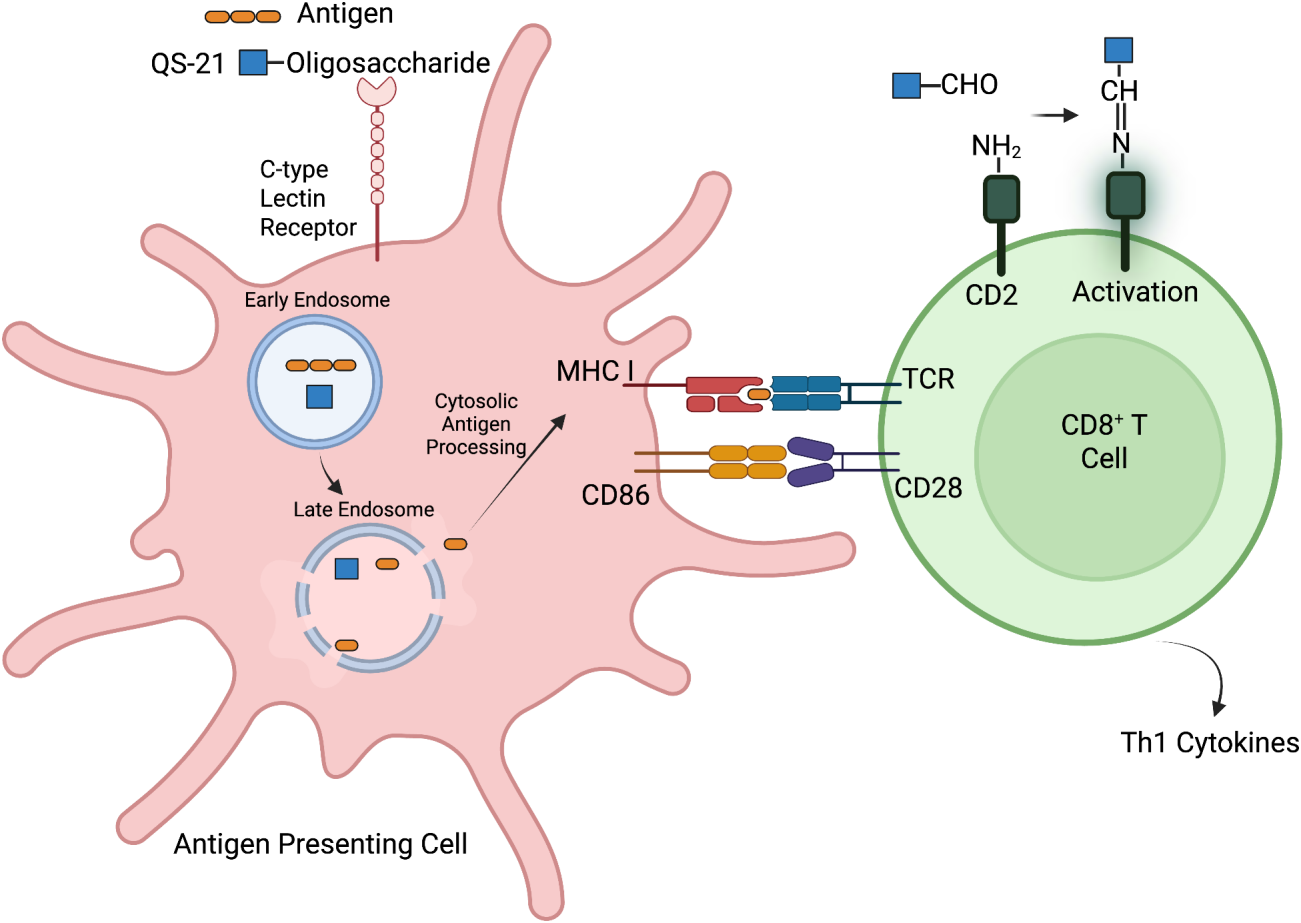
Proposed mechanism of QS-21-mediated enhancement of antigen cross-presentation and CD8^+^ T cell activation. While the specific receptor for QS-21 on antigen-presenting cells (APCs) is unknown, its oligosaccharide moiety may engage with C-type lectin receptors on the cell surface. Upon uptake, QS-21 promotes lysosomal membrane destabilization, facilitating antigen escape into the cytosol for processing via the endogenous pathway. This leads to peptide loading onto MHC class I molecules and surface presentation. In parallel, QS-21’s C-23 aldehyde group is hypothesized to form a Schiff base (imine) with the ϵ-amino group on the CD2 receptor of T cells. This interaction may mimic the co-stimulatory signal typically provided by CD80/86–CD28 binding, enhancing T cell activation and production of Th1 cytokines. Together, these mechanisms contribute to efficient priming of CD8^+^ T cells (78). Created with Biorender.com.

QS-21 is known to stimulate only the Th1 immune response in some studies (10), while others report that the adjuvant promotes a balanced Th1/Th2 stimulation (74, 75). Investigations into the mechanisms underlying T cell activation by QS-21 have indicated that specific chemical groups on the adjuvant play a role in enhancing adaptive immune responses. As noted earlier, the chemical structure of QS-21 contains a carbon number 16 ɑ-anomeric (C-16ɑ) hydroxyl group, an aldehyde group at carbon number 23 (C-23) and an oligosaccharide esterified at the carbon number 28 (C-28) carboxylic acid; the first oligosaccharide residue, D-fucose, is acylated at its 4-hydroxyl group with a 18-carbon pseudodimeric acyl chain (76–78). The C-23 aldehyde group on QS-21 is hypothesized to form an imine group or Schiff base with the ϵ-amino group present in CD2 receptor of T cells, mimicking the co-stimulatory signal delivered by the interaction between CD80/86 ligands on APCs and the CD28 receptor on T cells, thereby promoting T cell activation (**Figure 2**) (54, 77–79). Abrogation of the C-23 aldehyde group such as by its reduction to an alcohol, or modification with glycine, has been reported to result in a loss of adjuvanticity (77). In contrast, other studies with synthetic analogs suggest that the C-23 aldehyde group is dispensable, and it is the C-16ɑ hydroxyl group that is important for the adjuvanticity of triterpene saponin such as in echinocytic acid derivatives (80, 81). Further research is needed to clarify the role of these functional groups, as substitution of the C-23 aldehyde with alternative moieties has been found to impair the adjuvanticity of QS-21, even when the C-16ɑ hydroxyl group is retained (77, 81). Clarity on these interactions is critical to enable tailor-designed adjuvants with predictable immunogenic properties for future use.

Mechanistic insights into the structural features of QS-21 provide a foundational understanding of how this saponin may interact with immune cells to promote T cell activation. However, physiological relevance of these molecular interactions is best appreciated in the context of *in vivo* studies that evaluate functional outcomes such as cytokine production, T cell proliferation, and generation of polyfunctional T cell responses. Several preclinical and clinical studies have demonstrated that QS-21, both alone and as part of adjuvant systems like AS01, enhances antigen-specific adaptive immunity. For instance, responses following intramuscular immunization of mice with HIV recombinant protein gp120 showed significant enhancement in T cell proliferation and IL-2 and IFN-γ production with 10 μg of QS-21, as compared to immunization with the antigen alone. Such studies underscore the fact that QS-21 can modulate T cell function even in the absence of MPLA (58). In a study where humans were immunized with HbsAg, it was observed that AS01 adjuvants containing 50 μg (AS01B) or 25 μg (AS01E) of QS-21 and MPLA, respectively, induced the highest levels of CD4^+^ T cell response. These were the only adjuvant formulations observed to stimulate antigen-specific polyfunctional CD4^+^ T cells expressing IFN-γ, TNF-α and IL-2, when compared to other adjuvants like alum, AS03A and AS04 which contain only MPLA (82). Similarly, in a *Mycobacterium tuberculosis* vaccination study using the modified M72 antigen, the M72/AS01 combination produced higher levels of antigen-specific Th1 CD4^+^ cell responses characterized by polyfunctional T cells (expressing either IL-2, TNF-α, or IFN-γ), as well as elevated serum IFN-γ levels after booster vaccination (83). In a malaria vaccination study comparing AS01B and AS02A adjuvant systems with RTS,S, a recombinant fusion protein of HBsAg (S) and portions of circumsporozoite protein of *Plasmodium falciparum* (84), the RTS,S/AS01B group demonstrated greater vaccine efficacy and developed higher numbers of circumsporozoite protein-specific CD4^+^ T cells expressing two or more activation markers (IL-2, IFN-γ, TNF-α, or CD40L) compared to the AS02A group (85).

QS-21 has been shown to induce cytotoxic T lymphocytes (CTLs) in subunit antigen based vaccines like HIV gp120 (86), respiratory syncytial virus (RSV) fusion protein (87), and OVA (88), in mice systems. In one study, both young and elderly mice subcutaneously immunized with OVA and 5 μg of QS-21 developed a robust CD8 T cell response and showed an enhanced Th1 signature, in contrast to other adjuvants such as addavax, alhydrogel, and GPI-0100 (71). A combination of MPLA and QS-21 (each at 5 μg/mL), when added to a culture of HSV-2 infected human epidermal cells and autologous nonadherent PBMCs, increased HSV-2 immediate early protein specific CD8 T cell cytotoxicity by 47-68%, via enhanced IL-12 and IFN-γ production by the non-adherent PBMCs (89). The acyl group of QS-21 is crucial for CTL activation, as deacylated QS-21 fails to elicit this response against exogenous antigens. This suggests a role for the lipophilic acyl group in delivery of soluble protein antigens to the cytosolic cell compartments for processing by endogenous MHC class I pathway, resulting in CTL activation in a receptor-independent manner (76). The hydrophobic acyl group might interact with similar moieties of protein antigens, forming quasi-stable antigen-adjuvant complexes that might be targeted to APCs for further processing (76). The importance of the acyl group is further highlighted by the fact that QT-0101, a derivative produced by the deacylation of the fucopyranose from *Quillaja* saponins, predominantly elicits a Th2 immune response (33, 90).

In addition to the above factors, formulation and dose of QS-21 also influences T cell response. In combination with recombinant antigens, QS-21 promotes T cell activation, and co-administration with MPLA further enhances this response (58). The importance of TLR4 signaling in elucidating the adaptive response has been determined in a murine model using wild type and TLR-deficient C57BL/6J mice (57). In mice immunized intramuscularly with OVA and HBsAgs formulated with or without AS01, it was observed that TLR4 signaling was essential for optimal induction of Th1-skewed IgG2c antibody production and robust antigen-specific CD4^+^ and CD8^+^ T-cell responses, highlighting the importance of these pathways in adjuvant activity of AS01 (57).

Several studies have investigated the impact of co-formulation of QS-21 with MPLA on the quality and magnitude of cellular and humoral immune responses. Interestingly, combination of QS-21 and MPLA promotes a shift from Th2 to Th1 response; for example, mice intramuscularly immunized with HIV recombinant protein gp120 formulated with 25 μg of MPLA and 10 μg of QS-21, generated antigen-specific T cells that secreted increased levels of IL-2 and IFN-γ, along with reduced levels of IL-5, compared to those immunized with soluble antigen alone or with alum (58).

In evaluating the humoral response in this context, mice immunized with gp120 combined with QS-21 and MPL exhibited IgG2a as the predominant anti-HIV subclass in their sera, whereas IgG1 was the major subclass in mice immunized with alum-adsorbed antigen (58). In an influenza split vaccination and booster study, sCal—a split vaccine prepared from A/Cal H1N1 strain, was used to compare the adjuvants QS-21/MPLA, CWS (BCG cell-wall skeleton) and CPG/MPL (73). QS-21/MPLA adjuvanted sCal induced the highest IgG1 and IgG2c levels in C57BL/6 mice, and the highest IgG2a levels in aged BALB/c mice (73). The study also showed that addition of MPLA to QS-21 resulted in 4-fold increased induction of IgG2b and IgG2c antibodies compared to QS-21 alone in C57BL/6 mice (73). Moreover, QS-21 itself has also been reported to induce an increased Th1 immune response by promoting conversion of humoral response from IgG1 (Th2) to IgG2c (Th1), when compared to other adjuvants like addavax, alhydrogel and GPI-0100 (71). However, other studies have shown that QS-21 can promote a balanced Th1/Th2 immune response (75). For e.g, intramuscular co-administration of influenza antigen with 1.5 μg of QS-21 in mice generated a balanced Th1-Th2 response indicated by antigen specific IgG1 and IgG2c generation (75).

Observed variations in the type of immune response promoted by QS-21 can be attributed to several factors such as the nature of antigen, QS-21 dosage, formulation, and delivery routes. Indeed, a study showed that oral administration of QS-21 at doses below 25 μg in mice induced only IgG1 antibodies, indicative of a Th2 response, whereas administration of 25 μg or 50 μg resulted in a stronger IgG1 response along with the induction of IgG2b antibodies (91). Further increasing the dose to 200 μg led to the detection of IgG2b, IgG2c, and IgG3, in addition to igG1, indicating that QS-21 promotes a Th2 response at a lower dose and a mixed Th1/Th2 profile at higher doses (91). It has also been shown that human monocyte derived macrophages, when stimulated with MPLA/QS-21 either in soluble or oil-in-water emulsion formulations, predominantly generate a Th2 cytokine response. However, when the cells were stimulated with liposomal formulation of MPLA/QS-21, they exhibited a Th1-biased response with lower levels of IL-12p40 and IL-10 (92). Liposomal formulation of MPL/QS-21, as in AS01, is known to generate higher levels of antigen specific Th1 CD4^+^ response in comparison to oil-in-water emulsion of MPL/QS-21, as in AS02 (83, 85). Taken together, these studies highlight the importance of QS-21 dose and formulations on the generated immune phenotype.

### Memory response development

A study on RTS,S/AS01 malaria vaccine with a delayed fractionated dose regimen showed that AS01 played a key role in the induction of inducible co-stimulatory molecule (ICOS) expressing and IL-21 secreting circumsporozoite-specific peripheral T follicular helper (pT_FH_) cells, a cell type crucial for the formation and maintenance of germinal centers where B cells undergo affinity maturation (93). ICOS is associated with follicular recruitment, differentiation and function of T_FH_ cells whereas IL-21 is a cytokine critical for the development and maintenance of germinal center responses, which are essential for B cell maturation and memory formation (93). The presence of pT_FH_ cells in vaccinated individuals was strongly correlated with the ability to generate a robust and sustained B cell response after vaccination. Circumsporozoite-specific memory B cells were also induced following the second vaccine dose, and their numbers were higher and more persistent in the delayed fractionation dose group. Another study in humans with recombinant HBsAg with different QS-21/MPLA adjuvant formulations (liposomal formulation- AS01B and oil-in-water emulsion formulations—AS02B and AS02V), showed that all three vaccine formulations induced high and sustained antigen-specific CD4^+^ polyfunctional T cells expressing at least 2 markers among CD40L, IL-2, TNF-α, and IFN-γ and antigen-specific memory B cells up to 4 years post-vaccination (94).

In a non-human primate vaccination study evaluating the impact of QS-21-containing saponin/MPLA nanoparticle adjuvant on memory immune responses, rhesus macaques were immunized with an HIV envelope (Env) trimer protein with varying doses of QS-21 nanoparticles (25 µg, 50 µg, 200 µg, and 400 µg) (95). QS-21 was found to strongly promote antigen-specific memory T cell responses in a dose-dependent manner, with the highest dose inducing an 11-fold increase in CD40L^+^OX40^+^ CD4 memory T cells, a 15-fold increase in IFN-γ^+^ CD4 memory T cells, and a 9-fold increase in IL-21^+^ CD4 memory T cells compared to the lowest dose (95). The persistent presence of memory T cells at 24 weeks post-immunization, particularly in higher QS-21 dose groups is indicative of durable cellular immunity, essential for long-term vaccine efficacy.

In parallel with its effects on memory T cell responses, QS-21 robustly promoted antigen-specific memory B cell responses, with the highest dose resulting in a 6-fold increase in memory B cells compared to the lowest dose, and a 159-fold expansion of memory B cells post-boost (95). Furthermore, bone marrow plasma cells, critical for long-term antibody production, were 7-fold higher in the 400 µg dose group, demonstrating that QS-21 fosters long-lived humoral immunity. Enhancement of B cell memory was attributed to the ability of QS-21 to drive germinal center reactions in lymph nodes, ensuring high-affinity maturation and sustained titers of antibodies. Indeed, QS-21 has been reported to enhance B cell activation in germinal centers (71). Additional studies have shown that AS01B promoted antigen uptake by cognate B cells in dLNs and can promote their activation and metabolic activity (96). Importantly, QS-21 also plays a crucial role in promoting antigen-specific T follicular helper (T_FH_) cell responses, essential for B cell function. The highest dose led to a 3.6-fold increase in circulating T_FH_ (cT_FH_) cells and significantly enhanced germinal center T_FH_ (germinal center-T_FH_) activity, further amplifying B cell differentiation and affinity maturation (95). Mechanistically, activation of DCs by QS-21, promoted T_FH_ differentiation through IL-21 signaling, ensuring strong and long-lasting B cell response. Thus, QS-21 effectively enhances memory T and B cells, as well as T_FH_ cell responses in a dose-dependent manner, underscoring efficacy in inducing durable immunity.

Investigators have compared immunological effects of QS-21 combined with MPLA (in a liposomal formulation) against cationic liposome based adjuvant, CAF01 to assess the differences in T_FH_ cell polarization and long-term humoral immunity development (97). The QS-21/MPLA combination provided several immunological advantages, including the induction of a strong Th1-polarized germinal center T_FH_ (T_FH_1) response, marked by increased frequencies of CXCR3^+^IFN-γ^+^ T_FH_ cells (97). This polarization was closely associated with the development of durable and high-quality antibody responses. Notably, QS-21/MPLA promoted the generation of functional memory immune responses, as evidenced by significantly higher and sustained Env-specific IgG antibody levels that persisted for at least 30 weeks post-immunization (97). Following protein boosting, animals previously primed with QS-21/MPLA showed a robust recall of Env-specific antibodies, indicating effective reactivation of memory B cells.

Together, the above studies underscore the pivotal role of QS-21 in driving a robust and durable humoral memory response by enhancing memory B cell formation, promoting germinal center activity, and supporting T follicular helper cell responses. Its ability to shape both the quality and longevity of antibody responses highlights the promise of QS-21 as a potent adjuvant for long-term vaccine efficacy. However, despite extensive work on the mechanisms of action of QS21, the precise cellular and molecular processes via which the adjuvant effects are mediated remain largely unclear and interactions with specific receptors or immune mediators have not been unraveled. Further studies to comprehensively address the role of QS-21 in inflammasome and T-cell activation are also required. Perhaps the most significant remaining challenge is that with the use of different model systems, immune agonists, dosages, routes of entry, and modes of characterization—it is extremely challenging to integrate the findings on innate and adaptive mechanisms associated with QS-21 to derive a holistic understanding of the mechanism of action. We propose that an ensemble systems level study can help facilitate future design of adjuvants. Further, it is also important to systematically accrue data generated from the multitude of studies to enable future machine learning readiness, enabling design of new-to-nature adjuvants.

## Formulation

QS-21 has amphiphilic properties, essential for forming homogenous mixtures with soluble antigens in vaccine formulations. However, this presents a need to ensure formulation and encapsulation in a manner that promotes bio-efficacy and stability. Also, as noted earlier, QS-21 is often used in concert with other adjuvants to minimize toxicity and maximize efficacy. The impact of formulation—both combinatorial use and chemistry is discussed in this section.

### Combinatorial Immuno-stimulation

A promising strategy for enhancing the effectiveness of newly developed vaccines is the integration of multiple adjuvants into a single formulation. One of the most successful combinations is QS-21 with MPLA, as seen in AS01 (50). This combination leads to a more balanced Th1/Th2 immune response and stronger adaptive immunity (98), making it highly effective in vaccines such as the Shingrix (99) and malaria vaccines (100). The manufacturing process of MPLA produces a heterogeneous mixture of compounds, varying in the number of acyl chains, each of which may exhibit distinct adjuvant properties (101). Synthetic agonists in contrast, provide highly purified, single-component structures specifically optimized for human TLR4 activation. Recent research has explored combining QS-21 with synthetic versions like FP20, a glucosamine-based TLR4 activator with immunostimulatory potency similar to MPLA, featuring improved chemical stability and is obtained in an efficient and scalable 6-step chemical synthesis process (102). B6 mice, when immunized with OVA co-formulated with a combination of QS-21 variant and FP20 at a sub-optimal dose (10 µg each), showed a synergistic effect and higher antigen specific IgG titers than when either component was used alone (102).

Aside from AS01, AS05, and AS15 are also liposome based adjuvant platforms combining QS-21 and MPLA with either alum or CpG7909 respectively (103, 104). In a study that evaluated the efficacy of AS05 versus AS01B for RTS,S vaccine, AS01B showed better immunogenicity at the cellular level than AS05 (103). With regards to toxicity, however, AS15 combined with different tumor antigens in animal models was shown to demonstrate a favorable safety profile (104). Indeed, AS15-containing vaccines induced strong immune responses, with 100% seroconversion rates, and that adverse effects such as transient inflammation and mild systemic responses were reversible (104) in both rabbits and cynomolgus monkeys. These findings suggest that AS15 can be a promising immunostimulant for future vaccine formulations​.

Vaccine studies in murine models of RSV infection have previously shown that combination of recombinant (r) IL-12 adsorbed to alum was an effective adjuvant for the fusion (F) protein-based vaccines (105). However, instead of alum, which typically induces a Th2 response, vaccination with a suboptimal dose of QS-21 (8 µg) with F/rIL-2 elicited F-protein specific functional cell-mediated and humoral immune responses, demonstrated by enhanced neutralizing titers in the sera and antigen-dependent killer cell precursors in the spleen (106). Thus, QS-21 and rIL-12 form a potent adjuvant combination for eliciting the immune response against F-protein.

A HSV vaccine study identified that combination of QS-21 and CpG, significantly boosted cellular immunity, as evidenced by elevated levels of IL-2 and IFN-γ, and increased the activation of both CD4^+^ and CD8^+^ T cells compared to the alum-adjuvanted groups (107). Notably, the immune responses induced by this combination were comparable to those generated by mRNA vaccines, indicating its potency in subunit formulations (107). Another study compared the efficacy of a triple adjuvant combination of MPLA, CpG oligodeoxynucleotide, and QS-21 to enhance the immune response against the Cell-Traversal Protein for Ookinetes and Sporozoites (CelTOS) of *Plasmodium falciparum* in BALB/c mice (108). The triple adjuvant combination (MCQ: MPLA/CpG/QS-21) led to highest antibody titers, avidity, and long-lasting immunity, as well as enhanced Th1 responses (higher IgG2a and IFN-γ production) (108). MCQ-adjuvanted vaccines achieved up to 88% reduction in *Plasmodium* oocyst formation suggesting strong potential to block malaria transmission (108)​. The response was significantly stronger compared to single adjuvants, validating the synergistic effect of combining adjuvants with distinct immune modulation profiles.

Additionally, in a following study the authors also tested whether combining the *Plasmodium falciparum* CelTOS antigen with dual (Poly I:C + QS-21; PQ) or triple (MPLA+ Poly I:C + QS-21; MPQ) combination formulations were found to enhance immunogenicity and functional activity compared to single adjuvants (109). Investigators identified that mice immunized with the dual and triple adjuvant formulations developed markedly higher levels of total IgG and IgG subclasses, particularly the cytophilic IgG2a and IgG2b, as well as antibodies of higher avidity, compared to mice immunized with single adjuvants alone (109). IFN-γ and TNF cytokine levels were significantly higher in the PQ and MPQ groups, indicating robust Th1-biased responses (109). Functional assays confirmed that these adjuvant combinations led to greater transmission-reducing activity (TRA), with the dual adjuvant group (PQ) achieving the highest inhibition of oocyst development (84%)—a level comparable to the triple adjuvant group (MPQ) (109). The findings suggest that the dual QS-21 and Poly I:C formulation is as effective as the triple mixture, offering a simpler and potentially safer adjuvant strategy for optimizing CelTOS-based malaria vaccines.

Aside from infectious diseases, QS-21 has been found to be a potent adjuvant for cancer antigens. A study screened 19 different adjuvants for cancer antigens Mucin-1 (MUC1) peptide and GD3 ganglioside conjugated with carrier molecule keyhole limpet hemocyanin, and identified QS-21 to be the most potent adjuvant for lymphocyte proliferation, cytokine production, and antigen specific IgG and IgM titer induction (110). In a follow-on study, investigators identified five different adjuvant combinations with QS-21 (+CpG, +MPL, +bacterial nucleotide, +non-ionic block copolymer CRL-1005, +Titermax, and lastly Titermax+CpG) to be superior to QS-21 alone for induction of MUC1 and GD3 specific antibody responses (111). Overall, combination strategies are advancing the use of QS-21 in next-generation vaccines by identifying combinations of different immunostimulators that enhance antigen-specific immunity while minimizing toxicity. Future research is focused on refining these formulations for broader clinical applications.

### Chemical formulation

QS-21 has been formulated into various delivery systems to enhance its immunogenicity while mitigating toxicity and instability. One of the most widely used formulations involves liposomal encapsulation, as seen in the AS01 and AS15 adjuvant systems, where QS-21 and MPLA (and CpG DNA in case of AS15) are encapsulated into liposomes containing cholesterol and DOPC, to enhance antigen presentation and induce a strong Th1-biased immune response (2, 11, 112–114). These formulations have been successfully incorporated into vaccines such as Shingrix (herpes zoster) (99, 115) and RTS,S (malaria) (116), demonstrating efficacy in promoting both cellular and humoral immunity, while improving stability (117). Liposomal formulation of QS-21 in AS01B significantly reduces toxicity while preserving immunostimulatory properties. Free QS-21 causes hemolysis and local reactogenicity, including pain, inflammation, and tissue necrosis at the injection site (118–120). However, in AS01B liposomes, these adverse effects are mitigated due to the controlled release and stabilization within a phospholipid ensemble (121, 122).

The reason for reduction in toxicity is likely membrane sequestration of QS-21. Free QS-21 is amphiphilic, and consequently unstable in aqueous matrices. The significance of ensuring biochemical stability of amphiphiles in physiological matrices has been studied (123, 124). It interacts directly with cell membranes, leading to pore formation and lysis of red blood cells. However, in AS01B, QS-21 is incorporated into cholesterol-containing liposomes, which prevents direct contact with RBC membranes and reduces hemolytic activity (125). Free MPLA has pyrogenic effects in rabbits and humans (126, 127) and formulation into liposomes minimizes endotoxic properties (128, 129). Further, liposomal QS-21 in AS01B enables dose-sparing effects, meaning that a lower concentration of QS-21 is required to achieve the same immune response, further reducing potential toxicity. Studies comparing AS01B with free QS-21 formulations demonstrated that the liposomal delivery system lowered local reactogenicity while enhancing antibody titers and T-cell responses (130). Army Liposome Formulation with QS-21 is another liposomal platform made of saturated phospholipids like dimyristoyl phosphatidylglycerol and dimyristoyl phosphatidylcholine (DMPC), 55% mol of cholesterol compared to the total phospholipid, MPLA, and QS-21 (131, 132). This formulation is being extensively studied for use in vaccines targeting COVID-19 (133), HIV-1 (134, 135), malaria, (136) and *Campylobacter* diarrhea (137) in clinical trials.

Beyond liposomal platforms, other nanoparticle-based systems have also been developed to harness the adjuvant potential of QS-21. Another widely studied formulation is immune-stimulating complexes (ISCOMs), which are 40 nm open cage-like nanoparticles, composed of saponins like QS-21, cholesterol, and phospholipids (96, 138, 139). The particulate nature of ISCOMs allow efficient uptake by APCs (139), which can lead to robust CTL responses, making them particularly useful for both cancer and infectious disease vaccines. However, a challenge with the ISCOM system is that non-hydrophobic membrane proteins need to be modified for incorporation (22). The Matrix-M™ adjuvant system, an optimized version of ISCOMs, has the same structure and components as ISCOMS, but does not have any antigen incorporated (22, 140). This adjuvant system consists of two different nanoparticles mixed at a certain ratio, containing different *Q. saponaria* fractions with complementary properties (22, 52, 141). This gained attention due to successful application in the Novavax COVID-19 vaccine, where it induced CD4^+^ T cells with a Th1-biased response, increased neutralizing antibody titers compared to convalescent sera from hospitalized patients, and demonstrated an antigen dose-sparing effect in a phase 1–2 clinical trial (53).

Beyond liposomes and ISCOMs, other innovative nanoparticle-based formulations have emerged recently. Ionizable lipid-nanoparticles (LNPs) have been developed to formulate QS-21 based adjuvants. LNP-encapsulated QS-21 and cytosine-phospho-guanine (CpG) oligodeoxynucleotides (ODNs) has been used as an adjuvant system for varicella zoster virus (VZV) glycoprotein E (gE) subunit vaccine in mouse models (142). The LNP systems enhanced the synergistic effects of QS-21 and CpG and increased the VZV-gE-specific humoral response by ~2-fold, and antigen specific Th1 CD4^+^ T cell population by over 3.5-fold, compared to combinatorial use of CpG and QS-21 (142). Another group developed a LNP-based adjuvant formulation incorporating QS-21, among other immune-stimulatory components, to enhance the immunogenicity of a cancer vaccine (143). This iterative selection process identified that the combination of four adjuvants—CL401 (TLR2/7 agonist), motolimod (TLR8 agonist), 3D(6-acyl)PHAD (TLR4 agonist), and QS-21 (inflammasome activator)—synergistically enhanced cytokine secretion in APCs and boosted neoantigen-specific CD8^+^ T cell responses (143). Saponin MPLA nanoparticles based on QS-21 have been shown to be potent at eliciting germinal center B cell response, T_FH_, and antigen-specific antibody responses. However, the study showed that these particles induced transient increase in proinflammatory cytokines at a similar or lesser level than AS01B.

QS-21 conjugation with synthetic polymers or lipid-based emulsions to further enhance its stability and immune-activating properties has also been explored. Polymeric nanoparticles and emulsions can encapsulate QS-21 alongside antigens, allowing for synchronized delivery and improved immune response kinetics. Oil-in-water emulsions have been explored in combination with QS-21 to boost efficacy, particularly for viral vaccines (144). Furthermore, solid lipid nanoparticles (SLNs) and lipid-core nanocapsules (LCNs) have been examined as carriers for QS-21, providing a biocompatible and scalable solution for its incorporation into next-generation vaccines.

In summary, chemical formulation is critical to ensuring physiological function, biocompatibility, and controlled toxicity of amphiphilic adjuvants like QS-21. While liposomal formulations, ISCOMs, Matrix-M, and nanoparticles remain extensively studied as delivery systems for QS-21, emerging formulations such as aluminum-based adjuvants, polymeric conjugates, emulsions, and solid lipid nanoparticles offer additional avenues for optimizing its immunostimulatory potential. These advancements address the limitations of QS-21, such as its inherent instability and reactogenicity, while leveraging its ability to enhance both humoral and cell-mediated immunity. As new vaccine platforms continue to evolve, these diverse formulations ensure that QS-21 remains a cornerstone in the development of highly effective and durable vaccines across infectious diseases, oncology, and beyond.

Combinatorial adjuvants allow us to target cell mediated and humoral systems, stimulate innate immunity, and obtain tailored robust responses to antigens, which is a major advantage. However, our understanding of mechanisms of action of combinatorial formulations is inadequate. Therefore, it becomes necessary to tailor such formulations for each vaccine application, which is time consuming and expensive. In addition, combinatorial adjuvants present with a different toxicity profile than each adjuvant alone, and both localized (e.g., local inflammation) and systemic (e.g., fever, headaches) side-effects have been noticed in combination. A risk of breakdown of self-tolerance, resulting in a heightened sensitivity to autoimmune disorders has also been suggested. Beyond these gaps, there are significant regulatory hurdles to be addressed for each novel combination of adjuvants. Finally, formulation, dosing, route of entry, and process are as of yet not optimized even for single adjuvants, and present with significant heterogeneity in clinical studies. Such processes have to be re-optimized for each novel combinatorial adjuvant, which can be significantly challenging and delay product maturation. Streamlining this body of work—either to derive more comprehensive and curated information on mechanisms of action for various adjuvant combinations or modes to increase immunogenicity and decrease toxicity of individual adjuvants like QS-21—can truly advance this field significantly.

## Scalable production and manufacturing

**Figure 3:**
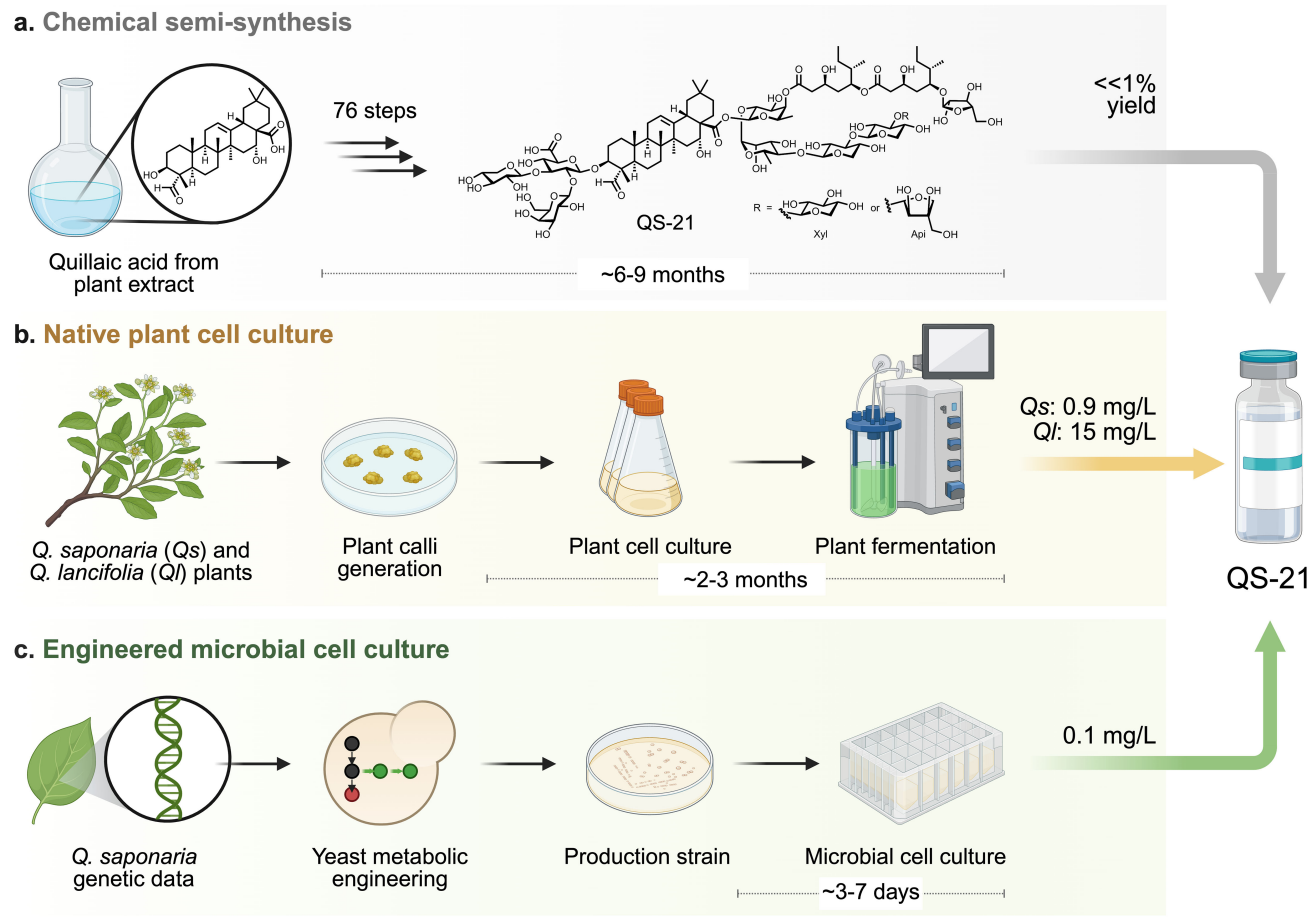
Strategies to replace the production of QS-21 in *Quillaja saponaria* bark include **(a)** chemical semi-synthesis, **(b)** native plant cell culture, and **(c)** engineered microbial cell culture. Schemes depict the generalized workflow to develop QS-21 production using each strategy as well as estimated production timelines and yields/titers. Created with Biorender.com.

The Chilean soapbark tree *Q. saponaria* can take up to 30 years to reach maturity, and harvesting its bark is potentially lethal to the tree, greatly limiting natural availability of QS-21. Further, extracting the adjuvant from harvested bark is labor-intensive and yields a limited amount of QS-21 (145), making large-scale production both costly and environmentally unsustainable. Furthermore, crude QuilA is a complex mixture of saponins, and isolating the active immunostimulatory saponin requires extensive purification that reduces overall yields (3). The slow growth of *Q. saponaria* and increasing global demand for vaccine adjuvants renders the supply chain for QS-21 particularly vulnerable to geopolitics, climate, pests, etc. QS-21 is a 65:35 mixture of two saponins with terminal C-28 apiose (QS-21-Api) and xylose (QS-21-Xyl) sugars, respectively; saponins isolated from *Q. saponaria* from the same local environment show variation in product distribution (6). Furthermore, QS-21 is chemically unstable (146), as the immunogenic acyl chain is prone to both transesterification within the attached D-fucose sugar as well as spontaneous hydrolysis at warmer temperatures and pH ≥ 7.4. Alternative strategies to produce QS-21 including chemical synthesis, *Q*. *saponaria* or *Quillaja brasiliensis (*currently recognized as *Quillaja lancifolia* [*Q. lancifolia*]) plant cell culture, and engineered *Saccharomyces cerevisiae* cell culture (**Figure 3**) (71), have been explored. In addition to obviating the need for isolating QS-21 from *Q. saponaria* or *Q. lancifolia*, these methods enable engineered (bio)synthesis to vary the chemical structure of QS-21 to access derivatives with improved bioactivity, reduced side effects, and increased chemical stability.

### Synthetic variants

The chemical total synthesis of QS-21 (both QS-21-Api and QS-21-Xyl) from quillaic acid was achieved through 76 steps with a negligible overall yield (**Figure 3a**) (147–149). To increase the stability of QS-21-based adjuvants, a semi-synthetic approach was developed that involved isolating the branched trisaccharide—triterpene portion (prosapogenin, QPS-CO_2_H) from *Quillaja* extracts and selectively modifying it (55, 80). One of the most significant changes was the replacement of the labile ester linkages in the acyl chain with more hydrolytically stable amide linkages, preventing spontaneous degradation. The semisynthetic method reduced the number of synthetic steps compared to the full chemical synthesis and created structural analogs with lower toxicity and comparable *in vivo* adjuvanticity. A truncated variant that lacked the entire branched trisaccharide domain attached to the carbon number 3 (C-3) of quillaic acid showed much less toxicity and elicited antibody responses similar to QS-21, leading to the discovery that this domain is not required for adjuvanticity (81).

A recent study synthesized 18 novel QS-21 analogs using oleanolic acid as the triterpene aglycone instead of quillaic acid, and by modifying four key structural domains: (A) the triterpene core, (B) the C-3 branched trisaccharide, (C) the C-28 linear tetrasaccharide, and (D) the acyl side chain (56). Modifications included substituting the C-3 branched trisaccharide for ketone or oxime functional groups at the C-3 position, replacing the C-3 branched trisaccharide with a simple glucose, and replacing the whole C-28 functionalization with a hydrolytically-stable bis-amide capped with glucose, maltose, or cellobiose (56). The authors performed biological evaluation of the hemolytic activity of these QS-21 analogs, an important factor in adjuvant safety, and showed that most exhibited significantly lower hemolysis rates compared to QS-21, with analogs L1–L8 demonstrating minimal hemolysis. However, some analogs, such as L12 and L15, still exhibited higher hemolysis rates, indicating that certain modifications may undesirably enhance hemolytic effects (56). The findings of this study suggest that these QS-21 analogs hold promise for developing safer and more effective vaccine adjuvants. Additionally, VSA-1, a semisynthetic saponin analog of QS-21, has been obtained from one step derivatization of *Momordica* saponin I (150), which was isolated from the inexpensive and widely available seeds of *Momordica cochinchinensis* Spreng (MC) (151). Synthetic QS-21 variants aim to retain the potent immune-activating properties of the natural compound while improving stability, reducing toxicity, and enabling scalable, cost-effective production.

### Plant cell culture

Plants form callus, undifferentiated masses of cells, in response to wounds or infection by pathogens; plant calli can be used for biotechnological applications because they are amenable to solid and liquid culture and can produce many of the same metabolites as their source plant (152–154). Importantly, plant cell culture enables the production of target metabolites (example, taxol) (155) from plant tissue in pest-free and defined environments that can control for nutrient availability, atmosphere composition, light exposure, etc. The homologous production of QS-21 via *Q. saponaria* plant cell cultures was recently achieved (**Figure 3b**) (71). Stem cuttings from mature *Q. saponaria* trees were harvested and grown on solid media. Callus tissue produced by these cuttings was transferred to liquid media and grown as a suspension. Using this approach 0.9 mg/L of QS-21 was obtained from ~2.6 kg fresh weight of plant tissue. Importantly, the QS-21 produced by *Q. saponaria* plant cell cultures and full trees were indistinguishable via analytical chemistry, *in vitro* cell bioactivity assays, and *in vivo* mouse bioactivity assays (71).

Recently, QS-21 production has been achieved using *Q. lancifolia* or Brazilian soapbark tree derived plant cell cultures, and this ranks among the best yields obtained to date from an entirely defined, non-tree-based *Quillaja* source (156). QS-21 was extracted from *Q. lancifolia* leaves using a simple aqueous extraction method followed by purification steps to enrich the saponin fraction. Cell culture suspensions were established from leaf-originated callus cultures and saponin aqueous extract was clarified and purified using solvent partitioning and chromatography to isolate bioactive saponins (156). The isolated bioactive saponin fraction, QB-90, contained QS-21 as one of its major components. The yield of QS-21 from this approach using unelicited cell culture suspension of *Q. lancifolia* was 1 mg/g dry weight (DW) or 15 mg/L of volumetric production. Elicitation techniques using salicylic acid further increased the yield to 3 mg/g DW (156). Overall, *Q. lancifolia* leaves represent another ecologically viable and sustainable alternative for the extraction of QS-21 and other saponins that exhibit structural and functional properties similar to those of *Q. saponaria* saponins (157). Together, plant cell culture of *Q. saponaria* or *Q. lancifolia* calli could enable the production of QS-21 at reduced environmental impact with lower risk of variability from environmental concerns. Genetic engineering of trees (158) or calli (153) could be used to access calli that produce increased amounts of QS-21 or new-to-nature QS-21 analogs; however, these approaches can be time-consuming and limited by low efficiency.

### Yeast cell culture

*S. cerevisiae* is a model eukaryotic microorganism for scalable production because it grows quickly in simple and inexpensive media, can be genetically engineered, and has been engineered to produce molecules [e.g., artemisinin (via semi-biosynthesis) (159–161)] at commercial scale for diverse applications (162). The complete biosynthetic pathway for QS-21 from *Q. saponaria* was recently discovered (163, 164), functionally characterized (78, 164) and refactored into yeast (*S. cerevisiae*) to achieve the heterologous microbial production of QS-21 from simple sugars (i.e., glucose and galactose) and salts (**Figure 3c**) (165).

*S. cerevisiae* was genetically engineered for the step-by-step construction of biosynthetic pathways to convert simple sugars (i.e., glucose and galactose) into QS-21-Xyl and QS-21-Api. This accomplishment required the upregulation of native yeast pathways as well as heterologous expression of 38 proteins sourced from six different organisms. First, the native yeast mevalonate and early sterol pathways were upregulated to establish a significant carbon flux towards 2,3-oxidosqualene. Second, this product was cyclized by a plant β-amyrin synthase and site-selectively oxidized by three plant cytochrome P450s (CYP450s) to yield the aglycone of QS-21, quillaic acid (QA). Third, nucleotide sugar synthesis pathways were engineered into yeast to produce seven uridine diphosphate (UDP) sugars (166, 167), and QA was site-specifically glycosylated by glycosyltransferases (GTs) resulting in the addition of a branched trisaccharide at C-3 (Xyl-Gal-GlcA-C3-QA) and a linear tetrasaccharide at C-28 (QA-C28-Xyl-Rha-Fuc-[Xyl or Api]). Fourth, an engineered type I polyketide synthase (PKS) from a fungus, two type III PKSs and two standalone ketoreductases (KRs) from plants produced a C_9_ acyl unit, and two plant acyl transferases attach this group iteratively to the glycosylated QA to create a dimeric C_18_ acyl chain. Lastly, a plant GT added Ara*f* to the acyl chain of the QS-21 precursor to yield the complete QS-21-Xyl and QS-21-Api molecule at titers of ~100 μg/L and ~30 μg/L, respectively. The successful microbial production of QS-21-Xyl and QS-21-Api was confirmed by NMR and high-resolution LC-MS/MS, where QS-21 produced by *Q. saponaria* and *S. cerevisiae* exhibit identical retention times, ^1^H chemical shifts, exact masses, and fragmentation patterns (165).

The biosynthetic production of QS-21 in yeast offers potentially significant advantages over other production methods. By eliminating the need for *Q. saponaria* trees, this approach reduces the environmental impact of QS-21 production and ensures a stable and renewable supply of QS-21. This method may scale with additional metabolic engineering to enable the future industrial production of QS-21. Owing to the genetic malleability of yeast, biosynthesis of QS-21 in this chassis may enable facile modification of the biosynthetic pathway and rapid access to different sets of QS-21 analogs compared to chemical synthesis. Screening the bioactivity of these sets of QS-21 analogs could allow the identification of analogs with improved stability and immunogenicity as well as reduced toxicity. Production in yeast is significantly faster than other production methods, taking only days to produce QS-21 compared to months for chemical synthesis, months for plant cell culture biosynthesis, and decades for natural *Q. saponaria* biosynthesis. Together, these advancements in the production of QS-21 in engineered yeast represent a breakthrough in vaccine adjuvant production, paving the way for sustainable and large-scale manufacturing of QS-21 and its analogs.

In summary, the potent adjuvanticity of QS-21 drives the ever-increasing demand for this critical saponin adjuvant by global vaccination campaigns. It is unlikely that traditional sourcing of QS-21, harvested from the bark of *Q. saponaria* trees, can continue to meet future demand without significant ecological and geopolitical ramifications. Several strategies have been explored to establish a secure supply chain of QS-21 and analogs thereof, including chemical (semi-)synthesis, engineered plant cell culture derived from *Q. saponaria/Q. lancifolia* calli, and engineered microbial production using *S. cerevisiae*. Semi-synthesis has provided numerous QS-21 analogs with comparable immunogenicity and abrogated toxicity; however, these methods rely on sourcing starting material from saponin-producing plants and may not be scalable to meet demand. Engineered plant culture holds great promise as a renewable platform for producing QS-21 in a scalable manner that eliminates the batch-to-batch variability observed in naturally sourced QS-21 and reduces production time, compared to trees, from decades to months. Engineered microbial production, currently accomplished in *S. cerevisiae*, has achieved titers comparable to traditional isolation while reducing production time to <1 week. Furthermore, the genetic malleability of *S. cerevisiae* renders it an ideal chassis for engineering the biosynthesis of QS-21 to produce new-to-nature analogs in a multiplexed, agile manner. Engineered biosynthesis is poised to address both the future demand for QS-21 as well as the challenge of rapidly exploring the chemical space of QS-21 analogs. However, at present, both plant cell culture and microbial production are at early stages in their respective technological developments. QS-21 sourced from these technologies will require extensive testing to satisfy requirements from regulating bodies. It also remains to be seen if these advancements are sufficiently disruptive and adequately scalable to supplant the traditional isolation of QS-21 from *Q. saponaria*.

## Conclusion and outlook

In this review, we comprehensively address the state of the science around QS-21, an immunomodulatory saponin, from bark to bench, highlighting key gaps and opportunities for improvement in three critical aspects: immunology, formulation, and manufacturing.

QS-21 is known to enhance vaccine efficacy *via* stimulation of both the innate and adaptive immune responses, making it an excellent candidate for use in vaccines and therapeutics targeting infectious diseases and cancer. The mechanism of action of QS-21 in activating innate immune cells such as DCs, monocytes, and macrophages is well-supported, but its influence on germinal center dynamics, T follicular helper cell responses, and long-term memory formation in humans remains under-explored. The bulk of existing evidence stems from murine or *in vitro* cell culture systems that do not always effectively translate to human systems. Thus, human systems immunology studies and clinical correlates of QS-21-induced immune memory are critically needed. The lack of standardization models, doses, routes of entry, formulation, and other aspects makes it challenging to cross-compare findings and effectively take advantage of the aggregate knowledge in this field of study. Looking ahead, further research into the structure-function relationship of QS-21, and analogous adjuvants, and their interactions with the immune system will be crucial for optimizing its adjuvanticity. Systematization of processes around adjuvant immunological characterization, together with curated data accrual can truly advance our understanding and simplify future needs. They can also enable future AI-readiness, making it possible to successfully design and develop new-to-nature adjuvants to suit specific and emerging needs in health and biodefense.

Clinical application of QS-21 has been limited by its chemical instability, toxicity, and hemolytic activity, necessitating continued refinement in its formulation and delivery. Recent advances such as chemical modifications and synthetic variants have addressed some of these limitations and yielded QS-21 derivatives with improved stability and reduced side effects. Encapsulation within lipid nanoparticles and liposomal formulations have enhanced the bioavailability of the molecule, while mitigating toxicity. Additionally, strategic combinations with other immunostimulants, such as MPLA and CpG have demonstrated stronger and more durable immune responses. We anticipate that investigating the immunogenicity of QS-21 with other Th1 stimulating pattern recognition receptor agonists like Poly I:C, R848, and others could further yield valuable insight and provide new adjuvant combinations for combating emerging disease. Future studies of interactions of QS-21 with PRRs and other conserved innate processes could help delineate additive effects to enhance vaccine efficacy.

Beyond QS-21, there are several saponin adjuvants that have been identified, like Quil A, QS-7, QS-17, and QS-18, broad use of which has been limited by toxicity, scalable sourcing, and other challenges. In efforts to overcome these limitations, alternative saponin-based delivery platforms have been developed. Among these, ISCOMATRIX™ represents a promising system that leverages the immunostimulatory potential of saponins in a scalable and stable format (140). ISCOMATRIX™, which has a core composition similar to ISCOMS but without any antigen (140), might be more cost-effective due to the use of saponin fractions instead of purified QS-21 and has been shown to be potent at inducing both Th1 and Th2 immune responses, resulting in the generation of both antigen-specific cytotoxic T cells and antibodies (168, 169). However, purification or synthesis of QS-21 enables precise control over dosing and composition and is important for identifying the structural motifs responsible for immunostimulation versus toxicity, thereby enabling the synthesis of next-generation adjuvants. ISCOMATRIX formulation may have less defined molecular characterization and more variability in composition. Ultimately, the choice between QS-21 and other saponin based formulations will depend on suitability for specific target population, regulatory predictability, safety, cost, and manufacturing feasibility.

The development of scalable and cost-effective production methods, including biosynthetic approaches, will also play a critical role in ensuring its widespread availability. As new vaccine technologies continue to evolve, QS-21-based adjuvants remain at the forefront of innovation, offering a potent and adaptable platform for enhancing immune protection in diverse populations.

Because of the limitations in current adjuvants, investigators have largely relied on combinatorial approaches which provide the required efficacy but minimize dose-associated toxicity of single adjuvants. Whereas these approaches have been effective at large, there is a significant need for design, optimization and development of such combinatorial adjuvants for each antigen formulation. Consequently, there is a growing need for adjuvants that can be tailored to specific pathogens and diseases; which in turn requires a deeper understanding of how adjuvants interact with the immune system; as well as approaches to minimizing toxicity. This presents the urgent need for platform approaches to quickly and effectively assess both innate and adaptive aspects of adjuvant immunology in a manner that ensures relevance and translation to human systems.

Beyond these factors, it is also important to consider that many adjuvants are natural products, scalable sourcing of which can prove both challenging and expensive. The three primary potentially renewable routes for QS-21 synthesis—total chemical synthesis, plant cell culture derived QS-21 and microbial biosynthesis—differ markedly in their readiness for large-scale industrial supply. Total chemical synthesis of QS-21 (76 steps) remains valuable for studying the structure-function relationship of the molecule and the generation of novel QS-21 analogs, however, is not feasible for cost-effective large-scale production due to low-yields. Plant cell culture has emerged as a pragmatic alternative to tree harvest, with recent work using *Q. lancifolia* cell culture suspension (unelicited) generating QS-21 yields of approximately 15 mg/L and indicating increasing readiness for integration into large-scale, industry-compatible production platforms. However, although promising and sustainable, QS-21 production from plant cell cultures will require significant capital and time to reach vaccine-scale supply, process optimization, and purification—challenges reminiscent of the long and capital-intensive taxol commercialization pathway. Microbial biosynthesis gained a major advance when the full biosynthetic pathway of QS-21 was reconstructed heterogeneously in engineered yeast. Although current titers remain far below the industrial scale, yeast offers rapid engineering cycles and access to already established large-scale fermentation systems. Substantial titer improvements via metabolic and protein engineering could make yeast-based production routes economically viable in the near future. Until such improvements emerge, a hybrid strategy—plant cell culture derived precursors combined with semi-synthetic modifications for stabilization or targeted functionalization—is a more pragmatic route for the generation of cost-effective QS-21. Looking forward, integrating considerations of cost, scalability, timeline to market, and environmental impact will be essential to identify the most sustainable and practical routes for QS-21 production.

Synthetic biology can greatly improve our ability to generate scalable production of adjuvants locally via fermentation-based methods. Such methods can also enable discovery of new-to-nature adjuvants, and using computational methods to interactively address toxicity/efficacy associated with biochemistry, we can rationally design, develop, and scale adjuvants for a variety of health applications effectively. In summary, we contend that the evolution of the state of the art in QS-21 development can truly revolutionize incorporation and advancement of novel disruptive technologies to advance adjuvant science in an unprecedented manner.

Whereas this review focuses on QS-21, we contend that the opportunities and challenges presented here mirror the requirements for adjuvant science broadly. Indeed, there is a critical need for adjuvants to enhance the immunogenicity of vaccine antigens and therapeutics. Historically, the development of adjuvants has been through trial and error; rather than by rational design. Whereas this has led to some effective adjuvants like alum, the inherent toxicity and uncharacterized mechanisms of action have limited the use of many other candidates. Thus, there is a need to advance methods to rationally design adjuvants, and to generate new-to-nature adjuvants. Emerging technologies such as discovery acceleration with AI/ML, or use of microbial manipulation tools such as synthetic biology can enable such tailored design of adjuvants. The use of these emerging and disruptive biotechnology methods can not only accelerate development but also enable broad and agile platforms for design and manufacturing, allowing for economic vitality and market resilience.
